# Uptake Characterization of Tumor Cell-derived Exosomes by Natural Killer Cells

**Published:** 2018-06

**Authors:** Ting HUYAN, Yongyong DU, Qiuping HUANG, Qingsheng HUANG, Qi LI

**Affiliations:** Key Laboratory for Space Bioscience and Space Biotechnology, School of Life Sciences, Northwestern Polytechnical University, Youyi Xilu, Xi’an 710072, Shaanxi, P. R. China

**Keywords:** Natural killer (NK) cells, Tumor cell lines, Exosomes, Biomarker, Uptake efficiency, Flow cytometry

## Abstract

**Background:**

Cancer is the leading cause of death in human disease and is a major public health problem around the world. Exosomes are a promising cancer biomarker and therapy target. Recent evidence demonstrate that tumor cells could inhibit natural killer (NK) cells’ immune surveillance function by releasing exosomes into tumor microenvironment. The intercelluar uptake of tumor cell-derived exosomes by NK cells is vital for using these exosomes in tumor diagnose and therapy. We aimed to investigate the efficiency of NK cell uptake of tumor exosomes.

**Methods:**

Exosomes derived from different tumor cells, RAW264.7 cells and NK cells were labeled by fluorescent dye and co-cultured with NK cells. The uptake rates of NK cells were observed by fluorescence microscope and analyzed by flow cytometry.

**Results:**

NK cells could take up more exosomes from themselves and cell lines originating from bone marrow. Epithelial cell lines can take up more exosomes from epithelial cells. There was no significant difference in uptake efficiency between Jurkat cells and RAW264.7 cells by NK cells, indicating that maybe the origin other than species affects the efficiency of recipient cell uptake of exosomes. Different tumor cells derived exosomes had different uptake efficiency by NK cells.

**Conclusion:**

There is certain pattern of NK cells uptake tumor exosomes, which provide important insights on how tumors affect NK cells and develop appropriate countermeasures. In addition, it can be also helpful to select and design proper exosomes as a drug carrier in future.

## Introduction

The emergence and spread of cancers such as gastric cancer, breast cancer and cervix cancer pose an enormous threat to global public health and the 5-yr survival rate of patients with cancer is very low (1). That tumor cell-derived exosomes are considered as a novel diagnostic biomarker and promising nano drug carriers for cancer therapy (2). Exosomes are extracellular vesicles (EVs) with a diameter of 30–100 nm (3). They are small phospholipid membrane-enclosed entities released by a wide spectrum of cell types and present in various body fluids (4). In cells, exosomes are released by exocytosis and contain specific cargoes (proteins, lipids, RNA, miRNA and ncRNA) (5, 6). Exosomes play a pivotal role in regulating biological activities in complex inter-cellular communication networks. Through receptor-mediated interactions, exosomes could directly act on target cells, transfer their contents from the host cells to the recipient cells and reprogram the functions of recipient cells (7). As membrane vesicles, exosomes have characters like low immunogenicity and high transport efficiency (8). In addition, they can protect miRNAs from RNase-induced degradation in cytoplast.

NK cells are kind of large granular lymphocytes, defined by the presence of CD56 and the absence of CD3 (CD56^+^CD3^−^) (9). Without prior immunization, NK cells have cytotoxic effects on oncogenically transformed cells and virus-infected cells and trigger subsequent adaptive immune responses by secreting numerous cytokines and chemokines (10). Furthermore, NK cells play an important role in tumor identification and surveillance, which makes them as a potential therapeutic strategy for many types of human malignancies (11). In clinical studies, NK cells are increasingly used as cytokine-activated killer (CIK) cells in anti-viral, anti-GvH (graft versus host) reaction, and anti-cancer trials (12, 13).

Currently, many studies have focused on the effects of tumor cells on NK cells via tumor-derived exosomes (TDEs) in the tumor microenvironment (14–16). Tumor cell-secreted exosomes can be taken up by various immunocytes, including T cells, regulatory T cells (Tregs), dendritic cells (DCs) and NK cells (17–19). The immune regulatory effects of TDEs are predominantly related to their inclusions. Clayton et al. found that soluble NKG2D ligands and growth factors in TDEs can down-regulate NKG2D expression in NK cells and inhibit their function (20). Since TDEs play important roles in regulating the function of NK cells, how NK cells uptake them and which types of TDEs are more likely uptaken by NK cells is an essential question to be addressed. By labelling a specific fluorescent dye (PKH-26), it was demonstrated that it is an active and specific process for target cells uptaking exosomes (21). However, to our knowledge, the uptake efficiency of NK cells for different TDEs has not been studied yet.

In the present study, we used PKH-67 (a fluorescent dye that labels lipids on membrane) to label the exosomes derived from different cells and analyzed the NK cells uptaking efficiency to elucidate the trends of NK cells uptaking of exosomes derived from different cells.

## Materials and Methods

### Ethics statement

The study has been approved by the Ethic Committees of Northwestern Polytechnical University. The ethics form and signed informed consent from all blood donors are provided in the supplementary materials.

### NK cell preparation

Peripheral venous blood (10 mL) was collected from healthy donors (n=10). The peripheral blood mononuclear cells (PBMCs) were collected according to the instructions of the lymphocyte separation solution (Haoyang TBD, No. LTS1077N). Primary human NK cells were separated from the peripheral blood mononuclear cells (PBMCs) by using NK Cell Isolation Kit (Miltenyi, No. 130-090-864) according to the instruction. Then, after labelling the CD56-PE (QuantoBio, No. A6803) and CD3-FITC (QuantoBio, No. A7032) monoclonal antibodies (mAbs) and their isotype-matched controls (IgG1-FITC/IgG2-PE) (Southern Biotech, No. 0102-02/0119-09), the purity of isolated NK cell was determined by flow cytometry (BD FACSCalibur, San Jose, CA, USA).

### Cell lines

The hepatoblastoma (HepG2, No. BNCC338070), cervix cancer (HeLa, No. BNCC337633) and breast carcinoma (MCF-7, No. BNCC337656) cells lines were purchased from the Wuhan Cell Institute of Chinese Academy of Sciences. They were maintained in DMEM cell culture medium (Gibco BRL, No.C12430500BT) supplemented with 10% fetal bovine serum (Exo-free FBS, ExoPerfectTM, No. EXOFBS50A-1), 10 mM nonessential amino acids, 1,000 IU/mL of penicillin, and 100 mg/mL of streptomycin in a humidified 5% CO_2_ atmosphere at 37°C. The myeloid leukemia cell line K562 (No. ATCC CCL-243), acute T leukemia cell line Jurkat (No. ATCC TIB-152), and mouse bone marrow derived macrophages RAW 264.7 (No. ATCC TIB-71) were purchased from the American Type Culture Collection (ATCC, Manassas, VA) and maintained in RPMI-1640 cell culture medium (Gibco BRL, No. C11875500BT) supplemented with 10% fetal bovine serum (Exo-free FBS, ExoPerfectTM, No. EXOFBS50A-1), 100 IU/mL penicillin, and 100 mg/mL streptomycin in a humidified 5% CO_2_ atmosphere at 37°C.

### Exosomes collection

The exosomes derived from each cell line were collected using exosomes isolation reagent (Total Exosomes Isolation Reagent, InvitrogenTM, No. 4478359). Culture media of each cell line and NK cells (1×10^7^ cells, cultured 24 h) were collected, and precipitation and centrifugation steps were performed according to the manufacturer’s instruction. After the last centrifugation, the pellets were resuspended in 1×PBS, ddH_2_O or specific dye buffer for different analyses.

### Exosomes identification

For particle size analysis, collected exosomes were diluted by 1×PBS, and measured by a Zetasizer Nano ZS (Malvern Instruments). The particle size and concentration of exosomes from four groups were measured 3 times. The ambient temperature was maintained at 23 °C–28 °C.

For SEM imaging, collected exosomes were resuspended in 2% paraformaldehyde aqueous solution and then diluted in ddH_2_O. Then, 5 μL exosome suspension was added to cleaned mica chips and air dried. Sample on the mica chips were imaged by scanning electron microscopy (SEM) (HITACHI S-4800, HITACHI) at 10 kV with a CCD camera (Gatan, Warrendale).

For flow cytometry assay, collected exosomes were stained by the exosomes-specific marker CD63 (BD, CD63-antibody-FITC, No. 557288) and CD81 (BD, CD81-antibody-FITC, No. 551108) monoclonal antibodies (mAbs) and their isotype-matched controls (IgG1-FITC) (SouthernBiotech, No. 0102-02). The expression levels of CD63 and CD81 were determined by flow cytometry (BD, Accuri C6).

### Exosomes quantification

The concentrations of exosomes derived from different cell lines were evaluated by BCA assay (BCA Protein Assay Kit, Beyotime, No. P0012-1).

### Exosomes staining

Exosomes were labelled with the PKH-67 Green Fluorescent Cell Linker Kit (Sigma-Aldrich, No. PKH67GL) according to the instructions. Exosomes were resuspended in 1 mL dye buffer. Then, 2 μL PKH-67 was added to and mixed with the exosomes solution for 5 min. Five mL 1% BSA (bovine serum albumin, Sigma, No. 9048-46-8) was added to the mixture to bind excess dye. Stained exosomes were washed with PBS by ultracentrifugation at 100,000×g for 2 h and diluted in complete culture medium in same quantity based on the result of BAS assay.

### Exosomes uptake assay

Expanded NK cells (1×10^6^) were added in to one well of a 12-well culture plate and then quantitated. Next, labelled exosomes derived from different cell lines were added to the NK cell wells, and NK cell wells with the same volume of culture medium served as controls. After 24 h co-culture, the NK cells were observed by inverted fluorescence microscope (Leica, Inverted Fluorescence Microscope, DM IL LED) and analyzed by flow cytometry. The positive rate and mean fluorescence intensity (MFI) of NK cells (which took up the labelled exosomes) were calculated according to the flow cytometry data.

### Data analysis

Statistical calculations were performed using SPSS 16.0 statistical software (IBM, New York, USA). The data are presented as the mean ± SD. The results were analyzed using analysis of variance (ANOVA). Multiple comparisons used the LSD test to evaluate the significant differences between the groups. Statistical significance was defined as *P*< 0.05.

## Results

### NK cell identification

**Fig. 1:**
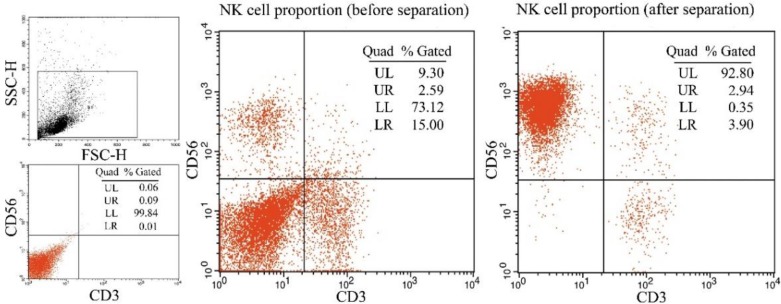
Pre- and post-expansion PBMCs were analyzed using flow cytometry PBMC were co-cultured with stimulating cells and harvested after 21-day of ex vivo expansion, all pellets were stained with CD56-PE and CD3-FITC mAbs and analyzed by flow cytometry. The percentage of NK cells (CD56+CD3−) in the PBMC population was tested. A: Pre-expansion PBMCs were analyzed by flow cytometry. The percentage of NK cells (CD56+CD3−) in the PBMCs was 9.30 %; B: Post-expansion PBMCs were analyzed by flow cytometry. The percentage of NK cells in the PBMCs was 92.80 %

After separated by using NK Cell Isolation Kit, the purity of NK cells (CD56^+^CD3^−^) was 91.05±3.31% compared to the 10.03±4.11% of PBMCs (before separation) (n=3, Fig. 1). Thus, the separated cells were identified as NK cells and were used in the subsequent experiments.

### Exosomes identification

**Fig. 2:**
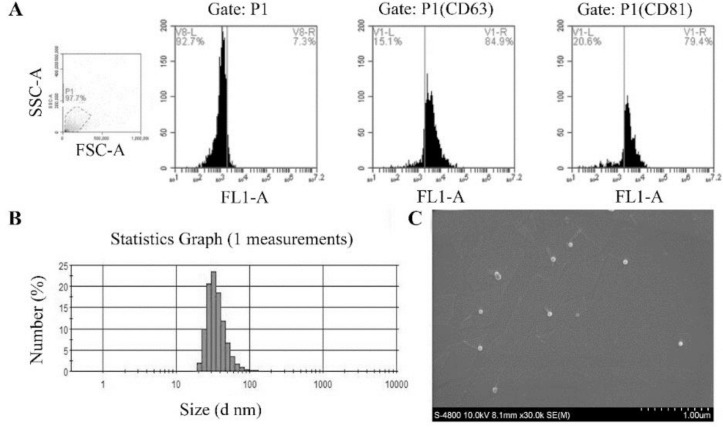
Identification of exosomes (illustrated by HepG2 exosomes) A: Flow cytometric analysis of the exosomes; exosomes were stained by CD63 and CD81 monoclonal antibodies and their isotype-matched controls. B: Particle size distribution of exosomes; exosomes were diluted by 1×PBS, and measured by a Zetasizer Nano ZS. C: Morphology analysis of exosomes by using SEM

The particle sizes of the exosomes (illustrated by HepG2 exosomes) were between 32.67 nm–164.2 nm (Fig. 2), and the mean size was 78.7 nm. Using the SEM, we showed that extracted exosomes present a sphere structure with a pit. The diameter of the exosomes was approximately 80 nm. Flow cytometry results indicated that these extracted exosomes express two specific exosomes proteins, CD63 and CD81. The positive rates of CD63 and CD81 were 84.4 ±7.23% and 93.7 ± 4.14%, respectively. These results showed that the extracted exosomes using exosomes isolation reagent had a uniform particle size and surface characteristics, which are the identification standards of exosomes (22).

### Exosomes uptake assay of NK cells

**Fig. 3:**
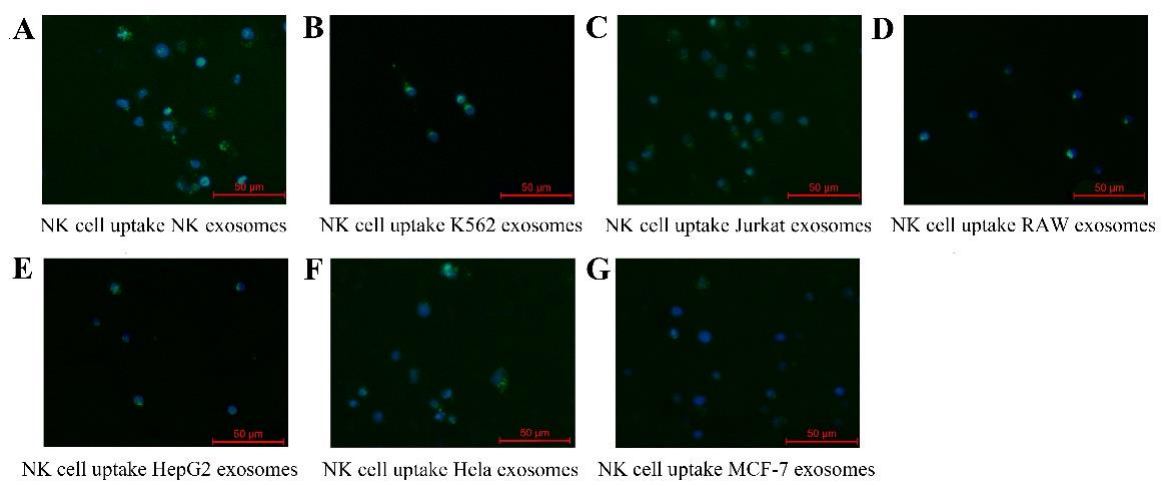
Exosomes uptake of NK cells (**A–G**): Representative fluorescence microscope images (merged) of NK cells co-cultured with PKH67 labeled exosomes derived from NK cells, K562 cells, Jurkat cells, RAW 264.7 cells, HepG2 cells, HeLa cells and MCF-7 cells respectively for 24 h. Blue is the DAPI stained nucleus

In order to examine whether exosomes derived from tumor cells could be uptaken by NK cells, exosomes were ladled with PKH-67 (green fluorescence) as described in the Methods section. The results from fluorescence microscopy showed that NK cells treated with all the exosomes demonstrated diffused fluorescence (Fig. 3). In particular, NK-exosomes and K562-exosomes exhibited a stronger fluorescence compare with other cell-derived exosomes.

Flow cytometry assay was also confirmed that NK cells have the highest uptake efficiency when they take up the exosomes from themselves, with a positive rate of 48.42 ± 2.05%. However, the positive rate of NK cells co-cultured with HeLa exosomes was 12.29±1.89%, and the data for HepG2 and MCF-7 cells were 17.91±1.25% and 11.37±1.8%, respectively. Compared to the three tumor cell lines, NK cells took up more K562 exosomes, and the positive rate was 28.39±2.55%. The uptake of K562 exosomes by NK cells was significantly higher than the other three groups. To determine if there were differ-ences in exosomes uptake efficiency among spe-cies, the uptake rates of exosomes from Jurkat cells and RAW 264.7 cells by NK cells were compared. The positive rate of NK cell uptake of the Jurkat cell group was 20.21±2.49% and for the RAW 264.7 cell group was 19.56±1.73%. All these results showed that there were different uptake efficiencies of NK cells on exosomes de-rived from different cells with different origins; however, there was no obvious difference be-tween the uptake rates of NK cells for exosomes derived from the same origin but different spe-cies (human and mouse).

**Fig. 4:**
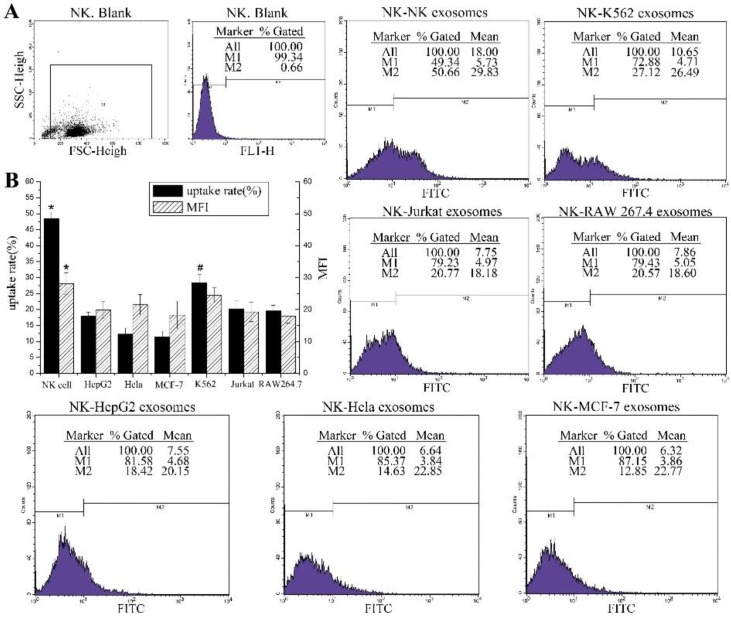
Exosomes uptake efficiencies of NK cells (**A**): Flow cytometric analysis of exosomes uptake efficiencies of NK cells; NK cells were co-cultured with the PKH67 labeled exosomes derived from each cell (NK cells, K562 cells, Jurkat cells, RAW 264.7 cells, HepG2 cells, HeLa cells and MCF-7 cells respectively) for 24h and analyzed by flow cytometry. (**B**): The uptake rates and MFI of each group are summarized in the bar graph. Each column represents the mean ± SD from four independent experiments. One-way ANOVA and LSD test, *: *p* value of NK group was less than 0.05 compared with the other groups (n = 4). #: *p* value of K562 group was less than 0.05 compared with the other groups (n = 4).

The MFI in each group was 28.06 ± 3.41 (NK cell), 21.5±3.21 (HeLa cell), 19.85±2.74 (HepG2 cell), 18.28±4.33 (MCF-7 cells), 24.5±2.32 (K562 cell), 19.24±3.01 (Jurkat cell) and 17.98±2.23 (RAW 264.7 cell). In contrast to the data on positive rate, except for the MFI of the NK cell group, which was significantly higher than the other groups, there was no significant change in MFI among the other groups. These results indicate that the uptake capacity of each NK cell was limited, and the origin or species of the exosomes does not affect the uptake capacity of each NK cell. After 24 h co-culture, NK cells uptake the fluorescently labelled exosomes and show green fluorescence which is detectable (Fig. 4).

### Exosomes uptake assay of tumor cells

**Fig. 5:**
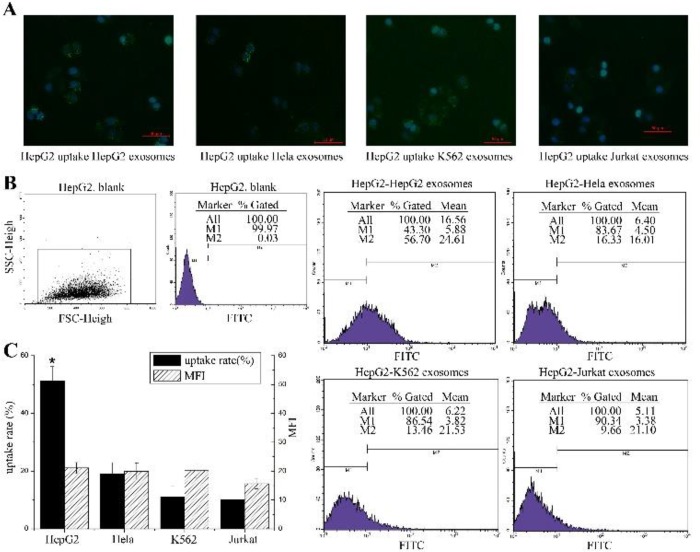
Exosomes uptake efficiencies by HepG2 cells (**A**): Representative fluorescence microscope images (merged) of HepG2 cells co-cultured with PKH67 labeled exosomes derived from HepG2 cells, HeLa cells, K562 cells, and Jurkat cells respectively for 24 h. Blue is the DAPI stained nucleus. (**B**): Flow cytometric analysis of exosomes uptake efficiencies of HepG2 cells; HepG2 cells were co-cultured with the PKH67 labeled exosomes derived from HeLa cells, K562 cells, HepG2 cells and Jurkat cells respectively for 24h and analyzed by flow cytometry. (**C**): The uptake rate and MFI of each group is summarized in the bar graph. Each column represents the mean ± SD from four independent experiments. One-way ANOVA and LSD test, *: *p* value of HepG2 group was less than 0.05 compared with the other groups (n = 4).

**Fig. 6:**
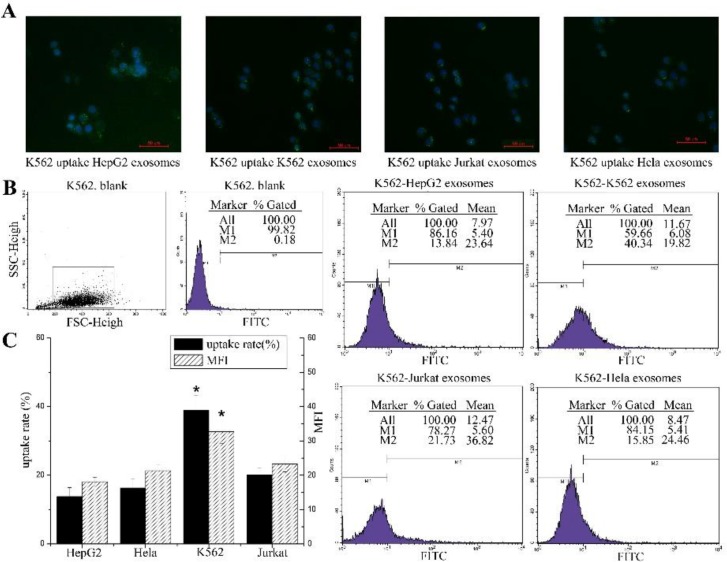
Exosomes uptake efficiencies by K562 cells (**A**): Representative fluorescence microscope images (merged) of K562 cells co-cultured with PKH67 labeled exosomes derived from HepG2 cells, K562 cells, Jurkat cells and HeLa cells respectively for 24 h. Blue is the DAPI stained nucleus. (**B**): Flow cytometric analysis of exosomes uptake efficiencies; K562 cells were co-cultured with the PKH67 labeled exosomes derived from HepG2 cells, K562 cells, Jurkat cells and HeLa cells respectively for 24h and analyzed by flow cytometry. (**C**): The uptake rate and MFI of each group is summarized in the bar graph. Each column represents the mean ± SD from four independent experiments. One-way ANOVA and LSD test, *: *p* value of K562 group was less than 0.05 compared with the other groups (n = 4)

To further explore the pattern of cell uptake of exosomes, the uptake capability of exosomes between tumor cells were also detected by microscopy and flow cytometry. HepG2 cells and K562 cells were used as the recipient cells. HepG2 cells treated with exosomes exhibited a spotted fluorescent pattern (Fig. 5A) while K562 cells treated with exosomes demonstrated more diffused fluorescence (Fig. 6A).

The flow cytometry results (Fig. 5B) showed that the uptake rate of HepG2 cells of their own exosomes was 51.2 ± 5.06%, and the corresponding MFI (Fig. 5C) was 21.12 ± 1.91. The uptake rates of HepG2 cells of exosomes derived from HeLa, K562, and Jurkat cells were 19.04±3.97%, 11.09 ± 3.84% and 10.06 ± 2.39%, respectively. The corresponding MFI values were 19.89±2.94 (HeLa), 20.18±2.4 (K562) and 15.67±1.8 (Jurkat).

When K562 cells were used as recipient cells, the data (Fig. 6B) showed that the positive rate of K562 cell uptake of K562 exosomes was 38.99±4.2%, and the MFI (Fig. 6C) was 32.68±3.36. The uptake rates of the other three cells were 13.79±2.59% (HepG2), 16.28±2.72% (HeLa) and 20.16±2.04% (Jurkat). Meanwhile, the MFI of the three cell lines were 18.1±1.32 (HepG2), 21.33±1.64 (HeLa) and 23.23±2.25 (Jurkat). These results also showed that the tumor cells were most likely to uptake their own exosomes and more efficiently uptaken the exosomes from tumor cells with the same origin with their own. For example, HepG2 take up more HeLa exosomes than K562 and Jurkat cell exosomes. There was a similar uptake pattern in K562 cells. Analogously, the MFI values in tumor cell were very similar among each group.

## Discussion

Cancer today is a public health concern of pandemic proportions, affecting more and more people every year. Immunotherapy is a promising way to overcome this public health problem. In this work, we provide a novel insight to investigate how tumor cells affect immune system via extracellular vesicles. Recently, exosomes and other extracellular vesicles have attracted much attention in biological research. In addition to their important role in intercellular communication, exosomes are represented as an important biomarker and vehicle for both diagnostic and therapeutic purposes in clinic. The shape, structure and inclusion of exosomes have been studied extensively. Especially their inclusions, including nucleic acids (mRNA, microRNA, circRNA, and long non-coding RNA) and proteins (cytokines, chemokines), play very important roles in regulating recipient cell functions. The mechanism of exosomes uptake crucially affect the responses of recipient cell, similarly to what has been demonstrated for the nanoparticles (23) and viruses(24). Therefore, considering the physiological features and functions of exosomes, some researchers have focused on how exosomes are taken up by recipient cells and the corresponding dynamic process. Based on current understanding, cells appear to take up exosomes by a variety of routes, including clathrin-dependent endocytosis and calthrin-independent pathways, such as caveolin-mediated uptake, macropinocytosis, phagocytosis and lipid raft-mediated internalization. The mechanism of cells take up exosomes may depend on proteins and glycoproteins on the surface of both the exosomes and recipient cells (25). Via receptor-mediated interactions, exosomes could directly act on target cells and transfer their content (cargo) from the host cells to the recipient cells (7). The uptaking of exosomes is an active and specific process, and storage of exosomes at conventional temperatures does not have any impact on its uptake process (21). In a recent study, Caponnetto et al. found the size and the preparation procedure of exosomes can affect their uptake efficiency by recipient cells. Their findings indicated that the exosomes extracted by polymer-based precipitation have smaller particle size distributions, which have increased cellular motility and faster uptaking by recipient cells. Different isolation methods lead to the different populations of particles with varying size distribution and cell motility, which may profoundly affect the exosomes therapeutic potentially (26).

We hypothesized that the efficiencies of recipient cells take up exosomes derived from different cells are different. As an important primary immune responder, NK cells play a key role in anti-tumor processes. However, tumor cells have also developed a series of mechanisms to inhibit NK cell activity and to escape immune surveillance. An increasing number of studies have shown that exosomes derived from tumor cells are important players in this process. How NK cells take up tumor exosomes and whether there are patterns in NK cells uptake of exosomes derived from different cells is thus of interest. By using fluorescence-labelled lipid dye, we analyzed the uptake efficiency of NK cells on exosomes derived from different cells. According to our results, most likely, NK cells are readily take up exosomes derived from themselves. The uptakes rate of NK cells on K562 and Jurkat cells exosomes are higher than the exosomes derived from HepG2, HeLa and MCF-7 cells significantly. K562 is human chronic myelogenous leukemic cell line, and Jurkat cells are an acute T cell leukemia lines. NK, K562 and Jurkat cells are derived from the myeloid system. However, HepG2, HeLa and MCF-7 are derived from hepatoblastoma (27), human cervix cancer and human breast cancer, respectively. Therefore, we hypothesized that the cells originating from same system may have more similar cell membrane compositions, and these cells more readily take up the exosomes from each other. Based on the origin, there is difference in uptake efficiency of exosomes between target cells. The same trend was also found in tumor cell lines. The uptake results from tumor cells showed that HepG2 cells take up more exosomes from themselves and HeLa cells, but the efficiency of HepG2 cell uptake of K562 cell exosomes is less than the former. Similarly, K562 cells uptake more exosomes derived from themselves and Jurkat cells and take up fewer exosomes from HepG2 and HeLa cells. By comparing the rate of NK cells uptake of exosomes from Jurkat and RAW 264.7 cells (mouse bone marrow derived macrophage), we observed that there was no significant difference between two cell lines, which indicates that maybe the origin rather than species is more important affecting the uptake rate of exosomes by recipient cells.

As an efficient and targeted natural nano carrier/vehicle, which avoids rapid clearance and toxicity associated with synthetic vehicles, exosomes are becoming more prevalent in efficient and targeted drug delivery and have gradually been used in clinical treatment. Therefore, elucidating the uptake efficiency of exosomes between cells would be helpful to choose suitable exosomes depending on the target cells used.

The size distribution of extracellular vesicles affected their uptake efficiency in recipient cells (28), and our results revealed that the origin of host cells might affect exosomes uptake efficiency by recipient cells. This may due to the cells with similar origins have more similar cell membrane components, and thus, exosomes uptake by each other is more efficient. However, more studies need to be done to further reveal the pattern of recipient cells uptake different exosomes and elucidate the underline mechanism. Meanwhile, the compositions of protein and lipid on the exosomes and recipient cells need to be analyzed, and corresponding inhibitors need to be used to identify the key players in the exosome uptake process. Furthermore, these studies were performed *in vitro*, and further study is still needed to carry out *in vivo* to simulate applicable situations.

## Conclusion

Different tumor cells derived exosomes had different uptake efficiency by NK cells. Specifically, NK cells could take up more exosomes from themselves and cell lines originating from bone marrow. Moreover, epithelial cell lines could take up more exosomes from epithelial cells. There was no significant difference in uptake efficiency between Jurkat cells and RAW264.7 cells by NK cells, indicating that maybe the origin other than species affects the efficiency of recipient cell uptake of exosomes. To elucidate the efficiency of NK cell uptake of tumor cell exosomes will help to determine how tumors affect NK cells and develop appropriate countermeasures. Furthermore, it will be helpful to select and/or design proper exosomes as a drug carrier for cancer therapy in future.

## Ethical considerations

Ethical issues (Including plagiarism, informed consent, misconduct, data fabrication and/or falsification, double publication and/or submission, redundancy, etc.) have been completely observed by the authors.
